# Dietary Iron Repletion following Early-Life Dietary Iron Deficiency Does Not Correct Regional Volumetric or Diffusion Tensor Changes in the Developing Pig Brain

**DOI:** 10.3389/fneur.2017.00735

**Published:** 2018-01-11

**Authors:** Austin T. Mudd, Joanne E. Fil, Laura C. Knight, Ryan N. Dilger

**Affiliations:** ^1^Piglet Nutrition & Cognition Laboratory, University of Illinois, Urbana, IL, United States; ^2^Neuroscience Program, University of Illinois, Urbana, IL, United States; ^3^Division of Nutrition Sciences, University of Illinois, Urbana, IL, United States; ^4^Beckman Institute for Advances Science and Technology, University of Illinois, Urbana, IL, United States; ^5^Department of Animal Sciences, University of Illinois, Urbana, IL, United States

**Keywords:** neurodevelopment, iron deficiency, pig, iron repletion, myelination, pediatric nutrition

## Abstract

**Background:**

Iron deficiency is the most common micronutrient deficiency worldwide and children are at an increased risk due to the rapid growth occurring during early life. The developing brain is highly dynamic, requires iron for proper function, and is thus vulnerable to inadequate iron supplies. Iron deficiency early in life results in altered myelination, neurotransmitter synthesis, neuron morphology, and later-life cognitive function. However, it remains unclear if dietary iron repletion after a period of iron deficiency can recover structural deficits in the brain.

**Method:**

Twenty-eight male pigs were provided either a control diet (CONT; *n* = 14; 23.5 mg Fe/L milk replacer) or an iron-deficient diet (ID; *n* = 14; 1.56 mg Fe/L milk replacer) for phase 1 of the study, from postnatal day (PND) 2 until 32. Twenty pigs (*n* = 10/diet from phase 1) were used in phase 2 of the study from PND 33 to 61, all pigs were provided a common iron sufficient diet, regardless of their early-life dietary iron status. All pigs remaining in the study were subjected to magnetic resonance imaging (MRI) at PND 32 and again at PND 61 using structural imaging sequences and diffusion tensor imaging (DTI) to assess volumetric and microstructural brain development, respectively. Data were analyzed using a two-way ANOVA to assess the main and interactive effects of early-life iron status and time.

**Results:**

An interactive effect was observed for absolute whole brain volumes, in which whole brain volumes of ID pigs were smaller at PND 32 but were not different than CONT pigs at PND 61. Analysis of brain region volumes relative to total brain volume indicated interactive effects (i.e., diet × day) in the cerebellum, olfactory bulb, and putamen-globus pallidus. Main effects of early-life iron status, regardless of imaging time point, were noted for decreased relative volumes of the left hippocampus, right hippocampus, thalamus, and increased relative white matter volume in ID pigs compared with CONT pigs. DTI indicated interactive effects for fractional anisotropy (FA) in the whole brain, left cortex, and right cortex. Main effects of early-life iron status, regardless of imaging time point, were observed for decreased FA values in the caudate, cerebellum, and internal capsule in ID pigs compared with CONT pigs. All comparisons described above were significant at *P* < 0.05.

**Conclusion:**

Results from this study indicate that dietary iron repletion is able to compensate for reduced absolute brain volumes early in life; however, microstructural changes and altered relative brain volumes persist despite iron repletion.

## Introduction

Iron is an essential micronutrient that is important for many physiological processes. Despite its importance in the body, iron deficiency remains the most pervasive micronutrient deficiency worldwide (1, 2). Moreover, iron deficiency appears to afflict all age groups, although it is more prevalent in children between birth and 5 years of age (3). Iron deficiency in early life is of great concern as this is a period during which brain development is highly dynamic and thus susceptible to alterations due to nutrition. Iron is specifically needed throughout neurodevelopment for neurotransmitter synthesis (4), myelination (5), and vascular development (6). Accordingly, reductions in dietary iron during the postnatal period greatly influence developmental trajectories. Research suggests that children who were iron deficient (ID) as infants exhibit delayed cognitive development later in life (7, 8), presumably due to structural alterations that developed in infancy and persisted despite iron repletion. These findings are supported by research in ID pigs, which suggests decreased spatial learning at 4 weeks of age (9) and 12 weeks of age (10). As such, there is a need to identify the structural changes that occur during periods of early-life iron deficiency and understand if any of these structural anomalies can be corrected by dietary iron repletion.

Iron is known to be important for neuronal growth and development. Research in 4-week-old pigs suggests that postnatal iron deficiency results in altered hippocampal gene expression for axon growth and guidance, as well as genes responsible for blood brain barrier integrity, angiogenesis, and hypoxia (11). Moreover, reductions in hippocampal iron content (9), alterations in hippocampal gray and white matter (12), and changes in hippocampal neurometabolites (12) all have been previously reported in 4-week-old ID pigs. In 12-week-old pigs that were provided ID diets from birth until 4 weeks of age followed by iron-replete diets until 12 weeks of age, altered hippocampal brain-derived neurotrophic factor (BDNF) profiles were observed, which suggests altered hippocampal plasticity despite iron repletion (13). In perinatally ID rodents, reduced hippocampal volumes and hippocampal subfield volumes have been reported (14). Analysis of rodent hippocampal cell cultures indicated decreased dendritic arborization and expansion as a result of induced iron deficiency (15). Moreover, this alteration in hippocampal dendritic morphology does not appear to be corrected in ID rats after receiving an iron-replete diet (16). Iron deficiency is also known to influence monoamine metabolism in subcortical brain regions, which may result in cognitive deficits later in life (17). While cellular and molecular mechanisms underlying the effects of iron deficiency on brain development have been extensively characterized in animal models, most of these approaches require invasive techniques.

The presence of iron in the brain is also required for proper myelination (5). Oligodendrocytes are rich in iron and are responsible for production of myelin in the brain (18). Iron is specifically needed for myelination because it serves as a cofactor for fatty acid synthesis (19), cholesterol utilization, and metabolic processes (18). Immunohistochemical analyses in rats that were exposed to perinatal iron deficiency indicated decreased myelin concentrations in the cerebellum and spinal cord (20) and a separate study reported reduced markers of oligodendrocyte activity (21). Additionally, postnatal iron deficiency in rats resulted in decreased concentrations of myelin basic protein and 2′,3′-cyclic nucleotide 3′-phosphohydrolase (CNPase), both of which increase as myelination occurs (22). The mechanisms that underlie changes in myelination due to iron deficiency appear to be well characterized; however, most of these markers also require invasive techniques for characterization. Moreover, most studies do not delineate which brain regions might be more susceptible to alterations in myelination due to iron deficiency.

To date, most studies focus on the influence of iron deficiency on hippocampal development; however, high concentrations of iron are found throughout the brain. Therefore, our study sought to characterize the global influence of iron deficiency on brain development and to assess if particular regions are more vulnerable at specific time points. Additionally, this study aimed to non-invasively characterize changes in brain development that occurred as a result of dietary iron deficiency up to 32 days of age, and if these changes could be corrected by 61 days of age, after a period of dietary iron repletion. In doing so, we used the pig because it is a superior model for assessing the influence of nutrition on neurodevelopment (23) and is a well-established model for early-life iron deficiency (9–13). This study is novel in that we employed a longitudinal design and non-invasive neuroimaging techniques to characterize the effects of iron deficiency at postnatal days (PNDs) 32 and 61. Findings from this study are poised to be clinically relevant as characterizations of neuroanatomical changes were assessed using non-invasive techniques, which are immediately translatable to infants in the first year of life. In this study, we hypothesized that dietary iron deficiency would alter brain development and a period of iron repletion may be able to recover particular aspects of brain development that were previously altered.

## Materials and Methods

### Animal Care and Use

All animal and experimental procedures were in accordance with the National Research Council Guide for the Care and Use of Laboratory Animals and approved by the University of Illinois at Urbana-Champaign Institutional Animal Care and Use Committee. Twenty-eight, naturally farrowed, intact male pigs were obtained from Carthage Veterinary Services and transferred to the University of Illinois Piglet Nutrition and Cognition Laboratory (PNCL) at PND 2. Per standard agricultural protocol, pigs were provided an intramuscular injection of a prophylactic antibiotic (0.1 mL of ceftiofur crystalline free acid such as Excede; Zoetis, Parsippany, NJ, USA) within 24 h of birth. Contrary to typical agricultural procedures, pigs on this study were not provided an iron dextran shot, as iron is the nutrient of interest in the experimental diet. Recent pig studies observed hippocampal transcriptome changes (24) and possible effects of iron overload (10) after iron dextran shot in the first few days of life, which further justify our decision to not provide iron dextran to any pigs. Upon arrival to PNCL on PND 2, pigs were stratified into one of the two experimental diets described below. Pigs were provided experimental milk replacer diets from PND 2 until PND 32 or 33 (phase 1), at which point both treatment groups were weaned onto the same series of weanling pig diet from PND 32 or 33 until PND 61 or 62 (phase 2).

For phase 1 of this study, 28 pigs were housed individually in custom pig rearing units (87.6 cm × 88.9 cm × 50.8 cm; *L* × *W* × *H*), which were composed of three acrylic walls, one stainless steel wall, and vinyl-coated metal flooring. Each caging unit was designed for pigs to see, hear, and smell, but not touch neighboring pigs. Pigs were allowed to physically interact with one another for approximately 15 min each day, and each pig was provided a toy for enrichment in their home-cage. Pig rearing environment was maintained on a 12 h light and dark cycle from 0800 to 2000 hours, with ambient temperature set at 26.6°C for the first 21 days of the study and gradually lowered to 22°C during the last 7 days of phase 1.

For phase 2 of this study, 20 pigs from phase 1 were transferred to the University of Illinois Veterinary Medicine Research Farm (VMRF) immediately following magnetic resonance imaging (MRI) at PND 32 or 33 and housed there until the end of the study. At VMRF, pigs were housed in floor pens (1.5 sq meters) and the rearing environment was maintained on a 12 h light and dark cycle from 0800 to 2000 hours, with ambient temperature set at approximately 22°C.

### Dietary Treatments

For phase 1 of this study, pigs (*N* = 28, *n* = 14/phase 1 diet) were provided one of the two dietary treatments with varying iron contents. The control diet (CONT) was formulated to meet all of the nutrient requirements of the growing pig and was formulated to contain 117.5 mg Fe/kg milk replacer powder. The ID diet was based off of the CONT diet; however, iron was only formulated to be supplemented at 7.8 mg Fe/kg milk replacer powder. Additionally, both diets were formulated to contain arachidonic acid (ARA) (2.08 g ARA/kg milk replacer powder) and docosahexaenoic acid (DHA) (1.04 g DHA/kg milk replacer powder). Milk replacer was reconstituted fresh daily with 200 g of milk replacer powder per 800 g water. Thus, formulated iron concentrations in reconstituted pig milk replacers were CONT (23.5 mg Fe/L milk replacer) and ID (1.56 mg Fe/L milk replacer). All pigs were provided *ad libitum* access to liquid diets from PND 2 until PND 32 or 33.

For phase 2 of this study, all pigs (*N* = 20, *n* = 10/phase 1 diet) were weaned onto the same series of iron-adequate diets, regardless of their phase 1 dietary iron status. Pigs were provided *ad libitum* access to standard complex diets (major ingredients including corn, whey, and soybean meal) and standard agricultural feeding practices were followed by sequentially switching from stage 1 diets, to stage 2 diets, to stage 3 diets, on PND 32, 41, and 50, respectively. During this phase of the study, all diets were formulated to meet all nutrient requirements of growing pigs (25), including iron. No zinc oxide, copper sulfate, or in-feed antibiotics were included in any diets.

### Magnetic Resonance Imaging

All pigs remaining in each phase underwent MRI procedures on PND 32 or 33 for phase 1 and again at PND 61 or 62 for phase 2, at the Beckman Institute Biomedical Imaging Center. For phase 1, 28 pigs (*n* = 14 per diet) were subjected to neuroimaging procedures and for phase 2, 20 pigs (*n* = 10 per phase 1 diet) were subjected to neuroimaging procedures. Imaging procedures were performed using a Siemens MAGNETOM Trio 3 T MRI, with a custom pig-specific 8-channel head coil at PND 32 and a human 8-channel head coil at PND 61. The pig neuroimaging protocol included three magnetization prepared rapid gradient-echo (MPRAGE) sequences and diffusion tensor imaging (DTI) to assess brain macrostructure and microstructure. Upon arrival to the imaging facility, anesthesia was induced using an intramuscular injection of telazol (50.0 mg of tiletamine plus 50.0 mg of zolazepam reconstituted with 5.0 mL deionized water; Zoetis, Florham Park, NJ) administered at 0.07 mL/kg BW, and maintained with inhalation of isoflurane (98% O_2_, 2% isoflurane). Pigs were immobilized during all MRI procedures. Visual observation of each pig’s well-being, as well as observations of heart rate, PO_2_ and percent of isoflurane were recorded every 5 min during the procedure. Total scan time for each pig was approximately 60 min. Upon completion of the scan, pig respiration and heart rate were monitored every 15 min until complete recovery from anesthesia. Imaging techniques are briefly described below.

#### Structural MRI Acquisition and Analysis

A T_1_-weighted magnetization-prepared rapid gradient echo (MPRAGE) sequence was used to obtain anatomic images of the pig brain, with a 0.7 mm isotropic voxel size. The following sequence-specific parameters were used to acquire T_1_-weighted MPRAGE data: repetition time (TR) = 1,900 ms; echo time (TE) = 2.49 ms; inversion time (TI) = 900 ms; 224 slices; field of view (FOV) = 180 mm; flip angle = 9°. Pig brains were manually extracted as previously described (26). All toolboxes described herein were available in SPM12, and Matlab R2015a was used for data processing. Once extracted, the “Coregister: Estimate & Reslice” toolbox was used to coregister individual brains to the Pig MRI Atlas (27). Next, the “Old Normalize: Estimate & Reslice” toolbox was used to transform individual pig brains into atlas space. The following parameters in Old Normalize were used for pig-specific data processing: template image (Pig MRI Atlas), bounding box (−30.1 −35 −28/30.1 44.8 31.5), voxel size (0.7). Finally, the “Deformations” toolbox was used to generate region of interest masks for volumetric assessment of individual brain regions in the pig MPRAGE space. In the deformation step, the estimated deformation from the Normalize step was used with a pushforward function, which was applied to all 19 regions of interest (defined by the Pig MRI Atlas), and the FOV image was defined as the individual pig’s skull-stripped coregistered brain image. A seventh degree interpolation was used and a binary mask of each region was generated. The fslstats-V function (FSL 5.0) was then used to estimate the volume of each individual brain region. In order to account for differences in absolute whole brain volume, all regions of interest were also expressed as a percent of total brain volume (%TBV), using the following equation: (region of interest absolute volume)/(total brain absolute volume) × (100), within subject.

#### Diffusion Tensor Imaging

Diffusion tensor imaging was used to assess white matter maturation and axonal tract integrity using a *b*-value = 1,000 s/mm^2^ across 30 directions and a 2 mm isotropic voxel. Diffusion-weighted echo-planar imaging (EPI) images were assessed in FSL for fractional anisotropy (FA), mean diffusivity (MD), axial diffusivity (AD), and radial diffusivity (RD) using the previously described methods (26). Assessment was performed over the following regions of interest: caudate, corpus callosum, cerebellum, both hippocampi, internal capsule, left and right cortex, thalamus, DTI-generated white matter, and atlas-generated white matter using a customized pig analysis pipeline and the FSL software package. For the purposes of this analysis, the Pig Brain atlas, generated from the same species, and previously reported by Conrad and colleagues were used (27). The diffusion toolbox in FSL was used to generate values of AD, RD, MD, and FA.

Masks for each ROI from the atlas were non-linearly transformed into the MPRAGE space of each individual pig and a linear transform was then applied to transfer each ROI into DTI space. A threshold of 0.5 was applied to each ROI, and the data were dilated twice. For each individual ROI, an FA threshold of 0.15 was applied to ensure inclusion of only white matter in the region of interest despite the mask expansion.

### Statistical Analysis

All researchers involved in this study (i.e., those performing daily procedures, data collection, and data analysis steps) remained blinded to dietary treatment identity until final data analyses had been completed. Data were analyzed by using the MIXED procedure of SAS 9.4 (SAS Institute, Cary, NC, USA). All longitudinal measures reported herein were obtained from pigs on both PND 32 and 61, thus all data were analyzed using a two-way repeated measures ANOVA (i.e., dietary iron status with postnatal day at time of neuroimaging acquisition as the repeated measure). Interactive effects were defined as an interaction between diet (CONT vs ID) and MRI day (PND 32 vs 61). Number of animals per treatment group was based on a power analysis using variability estimates from previous studies to detect differences with sufficient power of 80% at a significance of 0.05. Data were analyzed for outliers (defined as having a studentized residual with an absolute value greater than 3) and outliers were removed prior to statistical analysis. Significance was accepted at *P* ≤ 0.05. Data are presented as least-squares means with pooled standard errors of the mean (SEM).

## Results

### Pig Health Measures

Pig health measures were assessed at the end of each phase, and all outcomes are presented as means ± SD. At the end of phase 1, body weights of ID and CONT pigs were 6.24 ± 1.26 and 10.78 ± 1.45 kg, respectively, and at the end of phase 2 body weights of ID and CONT pigs were 17.05 ± 2.62 and 24.95 ± 2.40 kg, respectively. Average daily liquid milk intake for ID and CONT pigs during phase 1 was 0.93 ± 0.18 and 1.74 ± 0.24 L, respectively, and for phase 2, the average daily feed intake (as-is basis) for ID and CONT pigs was 0.73 ± 0.10 and 0.92 ± 0.13 kg, respectively. Hematocrit concentrations for ID and CONT pigs at the end of phase 1 were 14 ± 3 and 32 ± 3% packed cell volume (PCV), respectively, while at the end of phase 2, hematocrit concentrations in ID and CONT pigs were 35 ± 3 and 36 ± 2%, respectively. Hemoglobin concentrations for ID and CONT pigs at the end of phase 1 were 4.8 ± 0.9 and 10.86 ± 1.1 g/dL, respectively, while at the end of phase 2, hemoglobin concentrations for ID and CONT pigs were 11.85 ± 1.15 and 12.13 g/dL, respectively.

#### Absolute Brain Volumes

**Figure 1:**
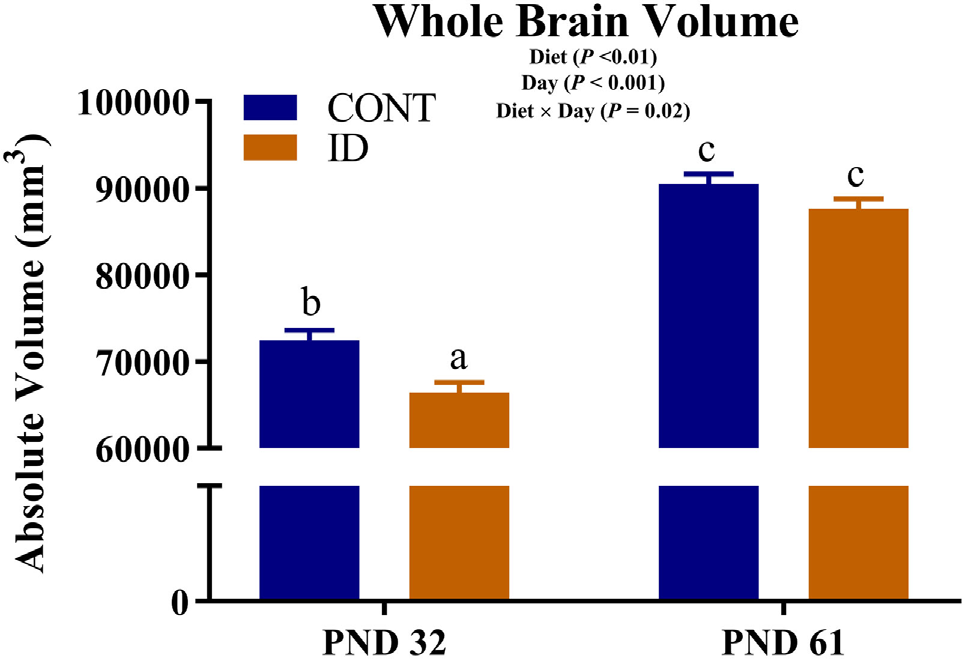
Iron deficiency influences whole brain volume at PND 32 and PND 61. Early-life dietary iron status influences pig absolute brain volumes over time. An interaction between dietary treatment and MRI day (*P* = 0.02), indicates differences (*P* < 0.05) in brain volumes at 32 days of age between CONT and ID pigs, but no difference (*P* > 0.05) in brain volume between CONT and ID pigs at 61 days of age. Abbreviations: CONT, control; ID, iron deficient; PND, postnatal day. ^a,b,c^Means without a common letter differ, *P* < 0.05.

An interactive effect of diet and MRI day was observed for whole brain volumes (*P* = 0.02), Figure 1. On PND 32, ID pigs exhibited smaller (*P* < 0.01) brain volumes compared with CONT pigs, and by PND 61 brain volumes were larger (*P* < 0.001) than volumes observed at PND 32, but were not different (*P* = 0.12) between ID and CONT pigs. Interactive effects were also observed for absolute volumes of the caudate (*P* = 0.01), gray matter (*P* < 0.01), hypothalamus (*P* = 0.01), internal capsule (*P* = 0.03), lateral ventricle (*P* = 0.05), olfactory bulb (*P* = 0.01), putamen-globus pallidus (*P* < 0.01), and right cortex (*P* = 0.02), Table S1 in Supplementary Material. Notably, these interactions are likely confounded by the difference in pig whole brain volumes, thus relative brain volumes are analyzed below. Also of note, a main effect of MRI day (*P* < 0.001) was observed for absolute volume of every brain region, indicating that all brain regions increased in size from PND 32 to 61, regardless of dietary treatment.

#### Relative Brain Volumes

**Figure 2:**
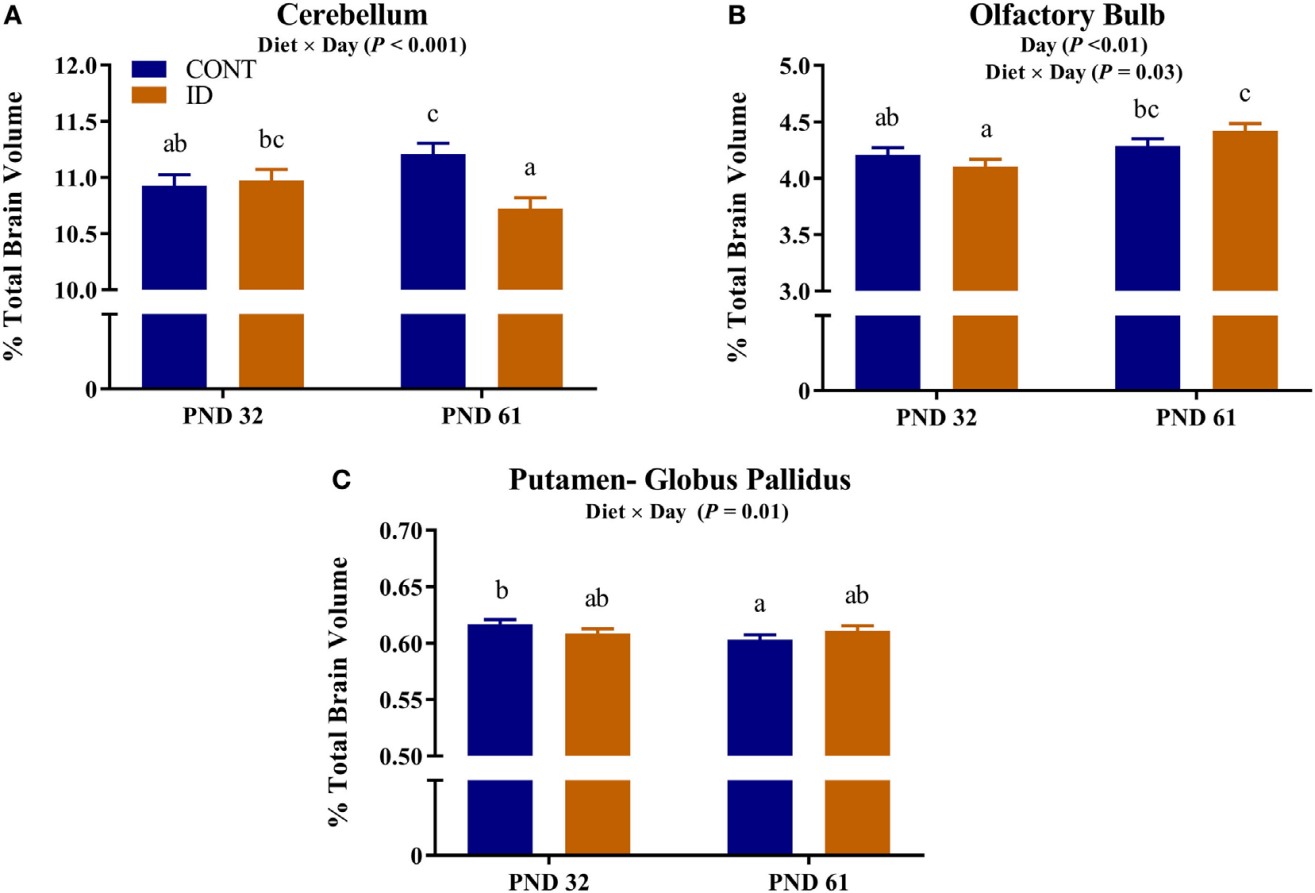
Iron deficiency influences relative brain volumes at PND 32 and PND 61. Early life dietary iron status influences pig relative brain volumes for specific brain regions over time. Interactive effects of dietary treatment and MRI day were observed for relative brain volumes (i.e., brain region as a percent of total brain volume) in the **(A)** cerebellum (*P* < 0.001), **(B)** olfactory bulb (*P* < 0.03), and **(C)** putamen-globus pallidus (*P* = 0.01). Abbreviations: CONT, control; ID, iron deficient; PND, postnatal day. ^a,b,c^Means without a common letter differ, *P* < 0.05.

**Figure 3:**
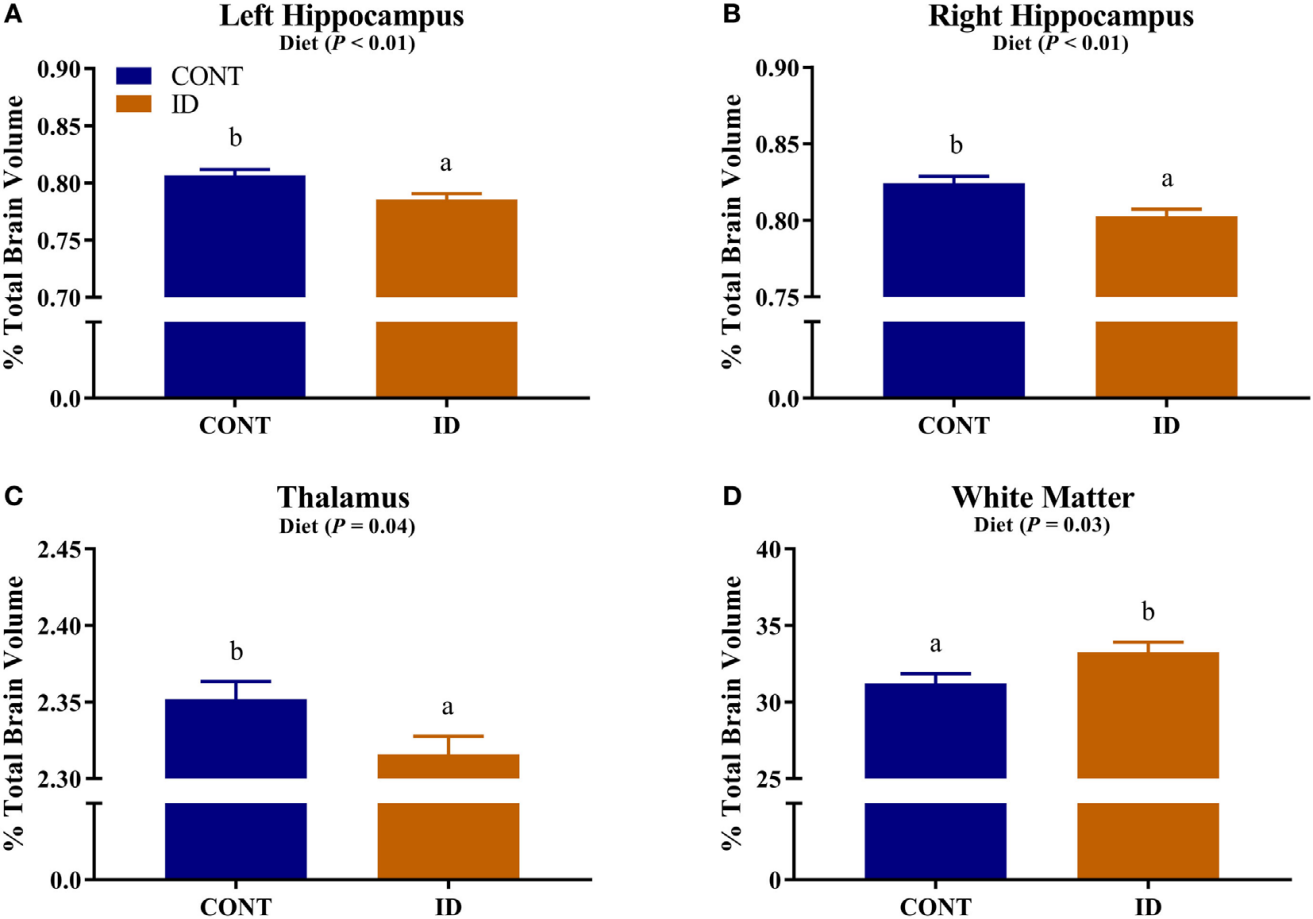
Iron deficiency influences relative brain volumes. Early life dietary iron status influences pig relative brain volumes for specific brain regions. Main effects of dietary treatment were also observed for relative volumes in the **(A)** left hippocampus (*P* < 0.01), **(B)** right hippocampus (*P* < 0.01), **(C)** thalamus (*P* = 0.04), and **(D)** white matter (*P* = 0.03). Abbreviations: CONT, control; ID, iron deficient. ^a,b^Means without a common letter differ, *P* < 0.05.

Due to observed differences in absolute whole brain volumes, all regions of interest were assessed relative to whole brain volume. Relative brain volume measures were generated by dividing the absolute volume for an individual region by the whole brain volume within subject and multiplying by 100, resulting in a percent of total brain volume measure. Interactive effects of diet and MRI day were observed for relative brain volumes in the cerebellum (*P* < 0.001), olfactory bulb (*P* = 0.03), and putamen-globus pallidus (*P* = 0.01), Figure 2. Relative brain volumes of the cerebellum indicated an increase (*P* < 0.01) in size from PND 32 to 61 in the CONT pigs and a decrease (*P* = 0.01) in relative size from PND 32 to 61 in the ID pigs. Analysis of the olfactory bulb indicated no change (*P* = 0.28) in relative size from PND 32 to 61 in the CONT pigs, whereas ID pigs exhibited an increase (*P* < 0.001) in relative size of the olfactory bulb from PND 32 to 61. The putamen-globus pallidus relative size decreased (*P* < 0.01) from PND 32 to 61 in the CONT pigs, but did not change (*P* = 0.60) during the same timeframe in ID pigs. Main effects of diet were also observed for relative volumes in the left hippocampus (*P* < 0.01), right hippocampus (*P* < 0.01), thalamus (*P* = 0.04), and white matter (*P* = 0.03), Figure 3. Notably, ID pigs exhibited decreased relative volumes in the left hippocampus, right hippocampus, and thalamus compared with CONT pigs. However, ID pigs exhibited increased white matter compared with CONT pigs. Main effects of MRI day were only observed for relative volumes of the caudate (*P* = 0.05), gray matter (*P* < 0.001), olfactory bulb (*P* < 0.001), and right cortex (*P* = 0.01), Table S2 in Supplementary Material.

#### Diffusion Tensor Measures

**Figure 4:**
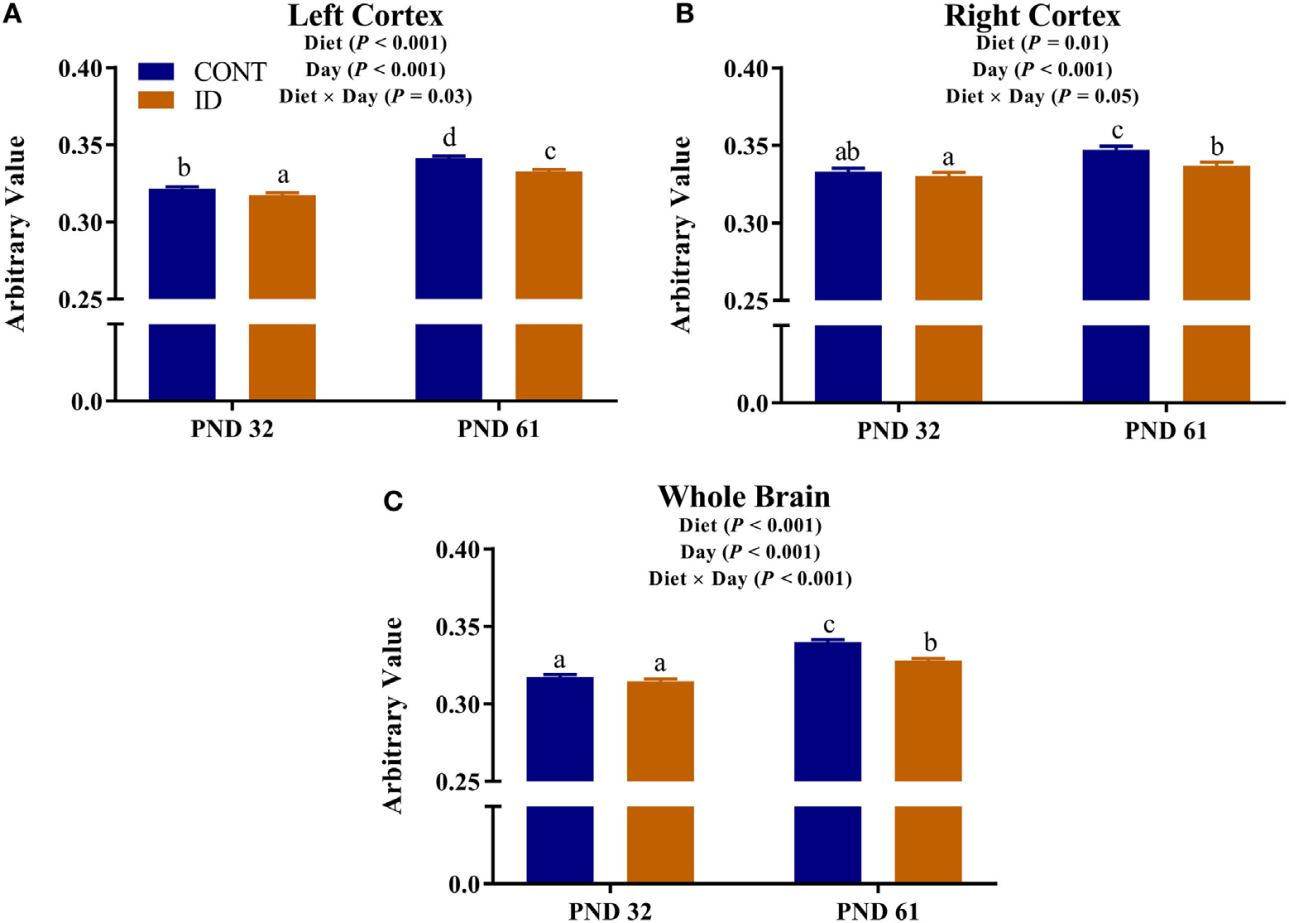
Iron deficiency influences FA at PND 32 and PND 61. Early life dietary iron status influences pig FA for specific brain regions over time. Diffusion measures of FA indicated interactive effects of dietary treatment and MRI day in the **(A)** left cortex (*P* = 0.03), **(B)** right cortex (*P* = 0.05), and **(C)** whole brain (*P* < 0.001). Abbreviations: CONT, control; ID, iron deficient; PND, postnatal day; FA, fractional anisotropy. ^a,b,c^Means without a common letter differ, *P* < 0.05.

**Figure 5:**
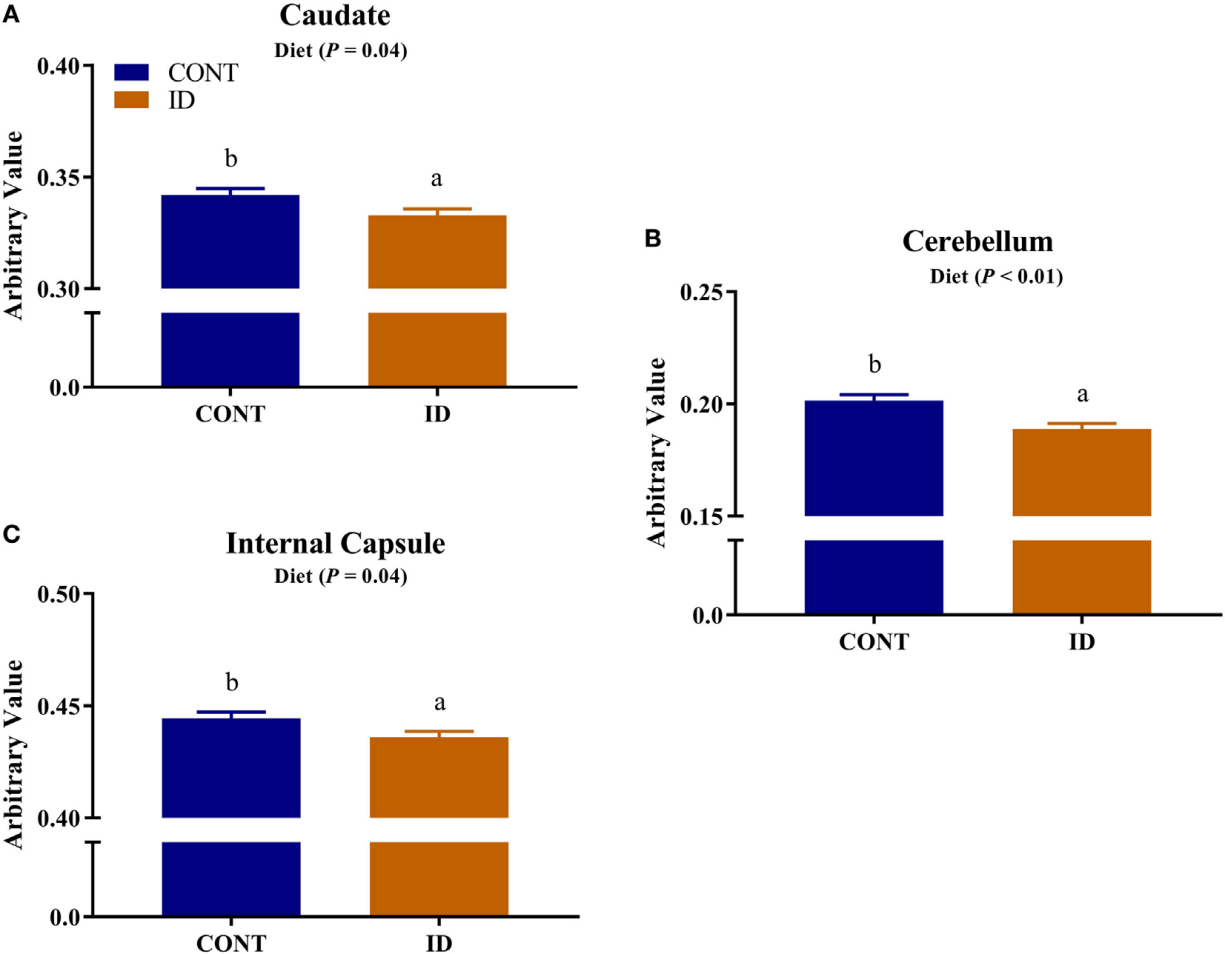
Iron deficiency influences FA measures. Early life dietary iron status influences pig FA for specific brain regions. Main effects of dietary treatment were observed for FA values in the **(A)** caudate (*P* = 0.04), **(B)** cerebellum (*P* < 0.01), and **(C)** internal capsule (*P* = 0.04), in all instances ID pigs exhibited decreased FA values compared with CONT pigs. Abbreviations: CONT, control; ID, iron deficient; FA, fractional anisotropy. ^a,b^Means without a common letter differ, *P* < 0.05.

Diffusion measures of FA indicated interactive effects of diet and MRI day in the left cortex (*P* = 0.03), right cortex (*P* = 0.05), and DTI-generated whole brain (*P* < 0.001), Figure 4. In the left cortex, ID pigs exhibited decreased (*P* = 0.03) FA values compared with CONT pigs at PND 32. By PND 61, left cortex FA values in both dietary treatments were increased (*P* < 0.001) compared with FA values at PND 32, but FA values for ID pigs remained lower (*P* < 0.001) than CONT pigs at PND 61. FA values in the right cortex and whole brain indicated no difference (*P* = 0.36) between ID and CONT pigs at PND 32, but by PND 61 ID pigs exhibited decreased (*P* < 0.01) FA values compared with CONT pigs. Main effects of diet were observed for FA values in the caudate (*P* = 0.04), cerebellum (*P* < 0.01), and internal capsule (*P* = 0.04), in all instances ID pigs exhibited decreased FA values compared with CONT pigs, Figure 5. Main effects for MRI day were observed for cerebellum (*P* < 0.001), corpus callosum (*P* < 0.001), and internal capsule (*P* < 0.001), Table 1. To verify our whole-brain FA findings, we applied a white matter mask, generated from the pig brain atlas, to the FA data for each pig. Our findings were consistent with the DTI-generated whole brain measures indicating an interactive effect (*P* < 0.001), Figure S1 in Supplementary Material.

**Table 1:** Fractional anisotropy (FA) measures (arbitrary values).^e^

Brain region	CONT		ID			*P*-value		
	PND 32	PND 61	PND 32	PND 61	SEM	Diet	Day	Diet × day
Caudate	0.340	0.344	0.327	0.339	0.00454	0.04	0.06	0.37
Cerebellum	0.192	0.211	0.178	0.199	0.00397	<0.01	<0.001	0.79
Corpus callosum	0.299	0.334	0.292	0.327	0.00440	0.16	<0.001	0.85
Internal capsule	0.433	0.456	0.421	0.450	0.00356	0.04	<0.001	0.21
Left cortex	0.321^b^	0.341^d^	0.317^a^	0.333^c^	0.00144	<0.01	<0.001	0.03
Left hippocampus	0.304	0.301	0.306	0.298	0.00515	0.91	0.19	0.43
Right cortex	0.333^a,b^	0.347^c^	0.330^a^	0.337^b^	0.00234	0.01	<0.001	0.05
Right hippocampus	0.300	0.302	0.292	0.305	0.00569	0.69	0.13	0.25
Thalamus	0.319	0.321	0.311	0.315	0.00542	0.14	0.58	0.87
Whole brain (from FA)^f^	0.317^a^	0.340^c^	0.315^a^	0.328^b^	0.00146	<0.001	<0.001	<0.001
Whole brain (from T_1_ scan)^g^	0.319^a^	0.339^c^	0.316^a^	0.329^b^	0.00148	<0.01	<0.001	0.01

*CONT, control; ID, iron deficient; PND, postnatal day; FA, fractional anisotropy*.

*^a,b,c,d^Means in a row without a common superscript letter differ, P < 0.05*.

*^e^Data presented as mean and pooled standard error of the means (SEM) for each dietary treatment group. Main effects of dietary treatment (Diet; CONT vs ID) and postnatal MRI day (Day; PND 32 vs 61) and the interaction between Diet and Day are presented*.

*^f^Whole brain FA values were generated from FA diffusion masks with a threshold of 0.15 to ensure only white matter was measured*.

*^g^Whole brain FA values were generated from T_1_-weighted white matter segmentation that was applied to the diffusion data*.

**Figure 6:**
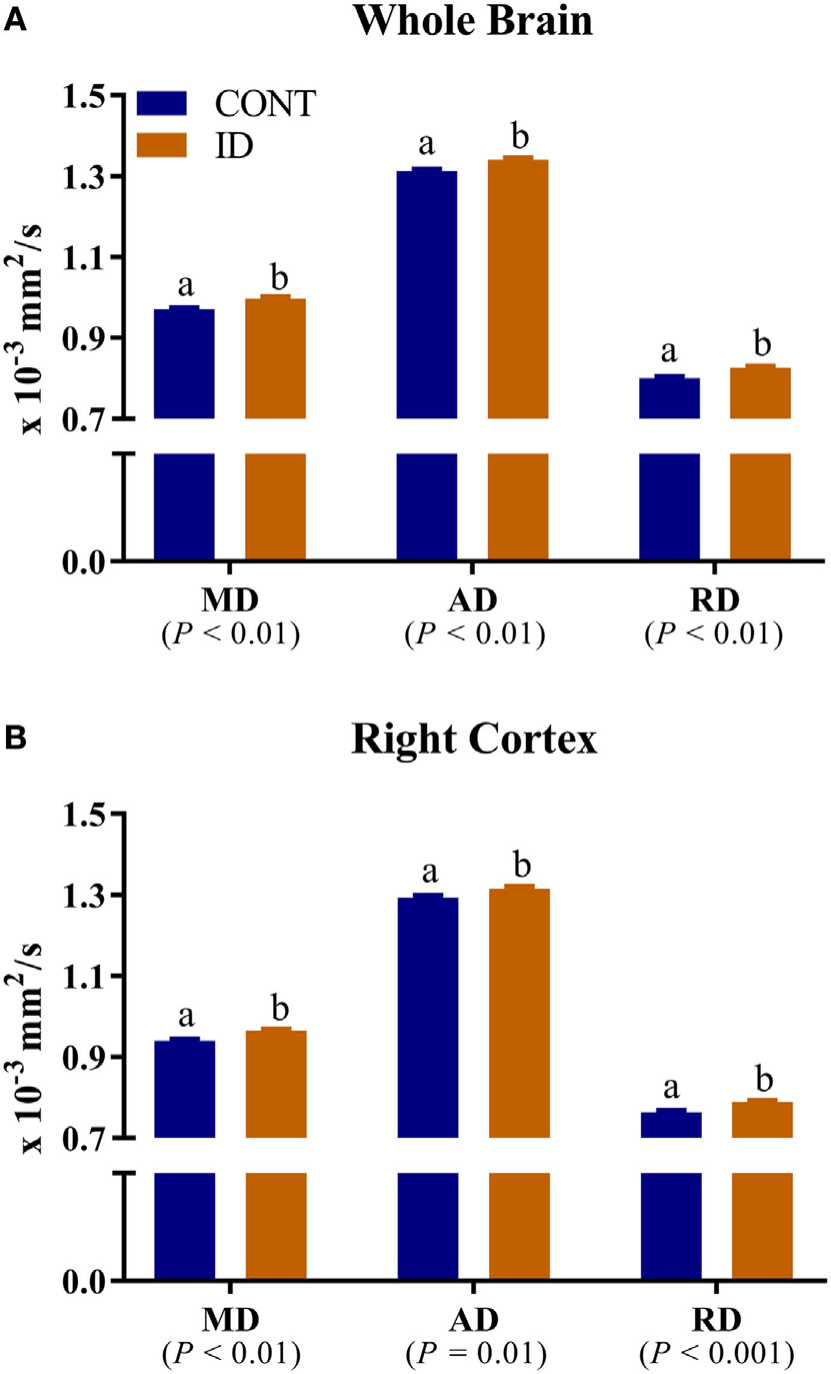
Iron deficiency influences mean, axial, and RD measures. Early life dietary iron status influences pig mean (MD), axial (AD), and radial (RD) diffusivity for the whole brain and right cortex. **(A)** A main effect of dietary treatment was observed for MD (*P* < 0.01), AD (*P* < 0.01), and RD (*P* < 0.01) in the whole brain. **(B)** A main effect of dietary treatment was observed for MD (*P* < 0.01), AD (*P* = 0.01), and RD (*P* < 0.001) in the right cortex. In all instances, diffusivity values were higher in ID pigs compared with CONT pigs. Abbreviations: Abbreviations: CONT, control; ID, iron deficient; MD, mean diffusivity; AD, axial diffusivity; RD, radial diffusivity. ^a,b^Means without a common letter differ, *P* < 0.05.

No interactive effects of diet and MRI day were observed for measures of RD. Main effects of diet were observed for RD in the left cortex (*P* = 0.03), Table S3 in Supplementary Material, right cortex (*P* < 0.001), and whole brain (*P* < 0.01), Figure 6. In all instances ID pigs exhibited increased RD values compared with CONT pigs. Main effects of MRI day were observed for RD measures in all analyzed regions (*P* < 0.05), except for the left hippocampus (*P* = 0.23), Table S3 in Supplementary Material. No interactive effects of diet and MRI day were observed for measures of MD. Main effects of diet were observed for MD in the right cortex (*P* < 0.01) and whole brain (*P* < 0.01), Figure 6. In both instances, ID pigs exhibited increased MD values compared with CONT pigs. Main effects of MRI day were observed for MD measures in all analyzed regions (*P* < 0.05), except for the cerebellum (*P* = 0.11) and left hippocampus (*P* = 0.11), Table S4 in Supplementary Material. No interactive effects of diet and MRI day were observed for measures of AD. Main effects of diet were observed for AD in the right cortex (*P* = 0.01) and whole brain (*P* < 0.01), Figure 6. In both instances, ID pigs exhibited increased AD values compared with CONT pigs. Main effects of MRI day were observed for all analyzed regions (*P* < 0.05), except for the cerebellum (*P* = 0.66), corpus callosum (*P* = 0.25), and left hippocampus (*P* = 0.05), Table S5 in Supplementary Material.

## Discussion

Iron deficiency influences structural brain development, which results in lasting functional deficits in animals and humans alike (19, 28, 29). However, it remains unclear which, if any, structural changes that result from early-life iron deficiency can be recovered by dietary iron repletion later in life. We used pig as a biomedical model to non-invasively assess the effects of early-life iron deficiency followed by dietary iron repletion on structural brain development. This study is novel because it utilizes non-invasive neuroimaging techniques to longitudinally characterize the effects of dietary iron status in the pig model, which provides clinically relevant information to be used in infant populations. To our knowledge, this is the first pig neuroimaging study to report longitudinal DTI measures, thereby providing insight into the timing of maturational events in the pig brain. Comparison of neuroimaging in human infant brains and pig brains suggests that one week of pig brain growth is roughly equal to one month of human brain growth (23). Thus, pigs that were imaged at PND 32 were approximately equal to a four-month-old infant and pigs that were imaged at PND 61 were equivalent to a six-to-12-month-old infant. Results from this study indicate that pig whole brain volumes are influenced by dietary iron status at PND 32, but are able to recover with dietary iron repletion by PND 61. Despite this apparent compensatory volumetric growth, analysis of relative brain region volumes and DTI measures indicate lasting microstructural changes, which may underlie known functional deficits later in life.

### Brain Volumes

Analysis of whole brain volumes revealed smaller brains in dietary ID pigs at PND 32 when compared with CONT pigs. Interestingly, after a 30-day period of dietary iron repletion, ID and CONT pig brain volumes were not different, thus suggesting compensatory brain volume growth in the ID pig brain. It has previously been reported that iron deficiency influences expression of genes related to axon guidance and expansion in the 4-week-old pig hippocampus (11). Thus, it is possible that neuron growth and expansion were inhibited during the period of dietary iron deficiency, but were no longer inhibited in ID pigs once they were provided an iron-replete diet. In pigs, volumetric whole-brain growth reaches the peak rate of development at 4 weeks of age (30), and it is possible that iron repletion during this dynamic period aided in compensatory volumetric expansion. These findings are in contrast to two ID pig studies in which whole brain volume at 4 weeks of age was not influenced by dietary iron status (9, 12). Notably, both of the previous pig studies provided an iron dextran shot to CONT pigs, whereas no pig in our study received an iron dextran shot. It is common agricultural practice to provide a newborn pig with 250 mg of iron dextran to prevent overt signs of iron deficiency prior to weaning because the sow’s milk is largely deficient in iron. It is possible that providing a surfeit amount of supplemental iron via an iron dextran shot may have influenced aspects of brain growth in the previous pig studies, as this has been shown to influence the hippocampal transcriptome (24) and possibly induce an iron overload in young pigs (10). Discrepancies between the studies may also be a result of improved neuroimaging hardware and techniques that provided a more sensitive assessment of structural changes reported in the current study.

Due to the differences in absolute brain volumes between dietary treatment groups, individual brain regions were assessed as a percent of total brain volume. Our analyses indicated volumes of the cerebellum, olfactory bulb, and putamen-globus pallidus were influenced by dietary iron status over time. Interestingly, cerebellar volumes were not different at PND 32, but by PND 61 dietary ID pigs exhibited smaller relative cerebellar volumes. Iron deficiency has been shown to delay cerebellar myelination in rodents (20), which may be the result of our observed volumetric differences. The putamen-globus pallidus region did not change over time in the ID pigs but decreased in relative size in CONT pigs from PND 32 to PND 61. It is unclear why there was no change in relative size in the ID pigs; however, this brain region is known to contain relatively high iron concentrations (31) and thus may be susceptible to changes in dietary iron during development.

Interestingly, relative volumes of the olfactory bulb were increased, relative to CONT, in ID pigs at PND 61, corroborating findings from a recent pig study, which reported increased gray matter in the olfactory bulb of ID pigs at 4 weeks of age (12). It remains unclear why the olfactory bulb is influenced by dietary iron deficiency, but it may be possible that this is a form of compensatory growth to aid the pig in acquiring iron through other dietary means. Iron deficiency was first observed in pigs in the 1920s when gestating sows were allowed to forage in fields up until a few days before farrowing (32). Once sows were moved inside and raised on concrete flooring for farrowing, foraging in the ground was no longer possible and their vegetable-based diets were largely deficient in available iron. Researchers noticed that pigs from these sows started to exhibit signs of iron deficiency by 3 weeks of age, and were able to correct these symptoms by supplementing iron in the diets of sows and pigs. Although not explicitly stated by McGowan and Crichton, this study highlights the foraging behaviors of pigs which allow them to maintain adequate iron stores through ingestion of exogenous sources, presumably dirt. Thus, in a case of iron deficiency, we speculate that the olfactory bulb may be preferentially developed to aid in seeking sources of iron in the environment. However, this hypothesis is purely speculative and future work should seek to elucidate the physiological relevance of this observed phenomenon. Additionally, it would be interesting to understand if this is a species-specific observation, or one that is relevant to all animals experiencing iron deficiency.

Regardless of age, pigs on the ID diet exhibited decreased relative volumes of the left and right hippocampi, as well as the thalamus. The observed effects in the hippocampi support previous work, which indicated prenatal, postnatal, and perinatal iron deficiency in rodents resulted in reduced hippocampal subfield volumes (33). In a separate study, Ranade and colleagues observed reductions in hippocampal volume in rodents whose dams were perinatally ID, and it is hypothesized that the observed differences are due to reduced glial cell proliferation (14). Rodent hippocampal cell cultures have shown reductions in dendritic arborization and growth due to iron deficiency further suggesting that iron deficiency can influence morphology of the developing hippocampus (15). Additionally, a recent pig study suggests dietary iron deficiency decreases hippocampal expression of genes responsible axon guidance and growth (11). Early-life iron deficiency followed by dietary iron repletion resulted in altered hippocampal BDNF and tyrosine receptor kinase B (TRKB) expression in pigs at 12 weeks of age, thus indicating lasting effects of an early-life ID diet on hippocampal development (13). Relative volumes of the thalamus were also decreased in pigs that were provided an ID diet early in life, and these findings support research that suggests the thalamus is sensitive to dietary iron status (19). In a 4-week-old pig model of iron deficiency, voxel-based morphometric analysis indicated decreased white matter and increased rates of diffusion, suggesting decreased myelination, in the thalamus of ID pigs (12), further supporting the sensitivity of this region to dietary iron deficiency in pigs.

Results from our study also indicate that relative volumes of white matter were increased in dietary ID pigs. At first pass, this finding might seem opposite of what has been reported, as iron deficiency is known to reduce myelination, which certainly contributes to white matter volume and maturation. However, when focusing on the absolute volumes of white matter in the CONT and ID pigs, we observed no differences between the groups at PND 32 or 61, yet whole brain volumes changed over time. Moreover, the absolute volume of gray matter increased to a larger extent in the ID pigs from PND 32 to 61 than it did in CONT pigs, suggesting that the observed whole brain compensatory growth is likely due to disproportionate growth in gray matter-rich regions. Thus, the larger relative white matter volumes in ID pigs may be driven by smaller absolute brain volumes at PND 32. Our results are in contrast to a previous pig study, which indicated region-specific decreases in white matter concentrations and FA measures of ID pigs (12). However, the results in our study assess absolute volumes relative to whole brain volume, whereas the study by Leyshon and colleagues (12) used voxel-wise comparisons to assess regional differences in white matter, regardless of brain size. Together, these findings of iron deficiency influencing total and relative regional brain volumes may be related to later life cognitive deficits observed in pigs, rodents, and humans (19, 28, 29).

### Diffusion Tensor Imaging

Diffusion tensor measures provide non-invasive measures of microstructural water movement and are related to axonal growth and myelinating events (34). FA is a diffusion measure that accounts for rate of water diffusion and direction of diffusion in tissue, and typically increases throughout development as myelination occurs and fiber coherence increases (35, 36). Our results indicate whole brain, left cortex, and right cortex FA values are influenced by dietary iron status over time. Measures of whole brain FA indicate both treatment groups exhibited increased measures from PND 32 to 61, indicating development occurred over time. No difference was observed between dietary treatments in whole brain FA value at PND 32, whereas decreased FA values were observed in ID pigs by PND 61 when compared with CONT pigs. It is interesting that at PND 61, whole brain volumes were not statistically different; however, diffusion measures suggest persistent microstructural changes despite a period of dietary iron repletion. These data support recent findings which indicate sensitivity of whole brain FA values in ID pigs (12), but it is important to point out that we did not observe differences until PND 61 (i.e., after providing iron-replete diets), whereas Leyshon and colleagues observed differences by 4 weeks of age. Analysis of left and right cortical FA values indicated increased FA values in both dietary treatments from PND 32 to 61; however, at both PND 32 and PND 61, ID pigs exhibited decreased measures compared with age-matched CONT pigs. These findings suggest that development occurred in both dietary treatment groups; however, dietary iron deficiency delayed cortical myelinating or tissue organization processes at each of the neuroimaging time points. A study of two-week-old infants suggests cortical FA values related to maternal dietary iron intake, suggesting sensitivity of cortical FA measures to dietary iron status (37). However, it should be noted that the study by Monk and colleagues (37) observed clusters of voxels where FA values inversely related to maternal reported iron intake. Our study did not assess maternal iron status nor FA values on a voxel-wise basis, instead our findings indicate decreased FA values averaged over left and right cortical regions in ID pigs. Comprehensive characterization of cortical diffusivity changes should be assessed in future studies to determine the normal trajectory of FA changes in pig brain development. Our results are novel because we comprehensively observe indications of decreased myelination and/or tissue organization despite observed compensatory volumetric growth after a period of dietary iron repletion. These data further suggest that particular aspects of neurodevelopment, might be more sensitive to the timing of early-life iron deficiency compared with other neurodevelopmental events such as brain volume growth.

Dietary iron deficiency also appeared to decrease FA measures in the cerebellum, caudate, and internal capsule, regardless of imaging time point. Indications of delayed myelination in the cerebellum of our pigs support observations in rodents, which suggest decreased myelination due to dietary iron deficiency (20). Because the decreased cerebellar FA values may indicate altered myelination, these findings may support our observations of decreased relative cerebellar volumes in ID pigs. A recent study in children suggests that increased iron content in the caudate positively relates to spatial IQ in 7- to-11-year-old children (38). Although our study does not report iron content of the caudate, decreased caudate FA values in ID pigs suggest the caudate is influenced by dietary iron status and these findings of early-life differences in caudate development may help to explain the observed differences noted in spatial IQ in children. Notably, pigs that were provided an ID diet for the first 4 weeks of life, followed by an iron–replete diet from 4-to-12 weeks, exhibited cognitive deficits even after iron repletion (10). Antonides and colleagues (10) observed decreased hippocampal iron content in the pigs at 12 weeks of age, which may have contributed to the cognitive deficits; however, our study suggests differences in the caudate may also underlie these cognitive deficits. The internal capsule is one of the earliest myelinating regions in infant development (39), and has been shown to be sensitive to dietary treatment in pigs (40, 41). A recent study observed decreased white matter, assessed through voxel-based morphometry, in the internal capsule of ID pigs (12). Therefore, the observed decreased FA values in the internal capsule of ID pigs support recent findings in an ID pig model and further suggest this brain region is highly sensitive to early-life nutrition. Notably, ID and ID-anemic infants were reported to exhibit delayed motor development (42), and it is known that both the internal capsule and cerebellum are involved in motor development. Thus, our diffusion data might suggest that delayed development as a result of iron deficiency in the internal capsule and cerebellum could underlie the observed motor development delays observed in infants.

Analysis of whole brain and right cortex MD, AD, and RD measures indicated increased rates of diffusion in the ID pigs compared with the CONT pigs, regardless of imaging time point. Leyshon and colleagues previously reported increased MD, AD, and RD in 4-week-old pigs; however, their observations were located in the hippocampi and thalamus (12), rather than the whole brain and right cortex as we observed. Extensive research in rodent models of early-life iron deficiency suggests lifelong alterations in myelin fatty acid profiles and gene expression for myelin basic protein, despite iron repletion later in life (19). Moreover, it is speculated that iron uptake may be influenced in preoligodendrocytes and oligodendrocytes (19), thereby attenuating myelination throughout development, which would support our findings, thus suggesting reduced myelination in particular brain regions. As described previously, absolute volumes of white matter were not different between treatment groups at PND 32 or PND 61, which might suggest that oligodendrocytes are present. However, as supported by our diffusion data, the mere presence of oligodendrocytes is not necessarily enough for myelination to proceed, as evidenced by lower FA values in previously-ID pigs at PND 61. Future work should seek to quantify iron content in the brain regions that are reported to be influenced by dietary iron deficiency and elucidate whether the physical presence of iron may be contributing to these observed changes in diffusion measures. Further characterization of brain iron content will help to illuminate the mechanisms though which dietary iron deficiency influences brain myelination, as well as characterization of oligodendrocyte maturation throughout the myelination process in the growing pig.

### Longitudinal MRI Assessment

A notable strength of this study is the implementation of a longitudinal dietary intervention in which brain development was measured at two time points. To our knowledge, no other neuroimaging study has comprehensively assessed changes in volumetric and diffusion measures over time in the pig. In doing so, this study provides insight into the growth and development of specific brain regions, and can therefore be used as normative data to which other studies can be compared when using the pig as a biomedical model. Analysis of absolute brain volumes and regions within the brain indicated all brain regions increased in size from PND 32 to 61. When assessing regional brain growth relative to whole brain volumes, the proportion of gray matter in the brain decreased and the olfactory bulb exhibited increases as a proportion of total brain volume. Assessment of diffusion tensor measures indicated region-specific FA values increased from PND 32 to 61, which supports observations in human neurodevelopment (35, 36). Future studies should seek to characterize changes in DTI measures at multiple time points throughout pig development. In doing so, researchers will be able to better identify particular developmental processes occurring in the brain (i.e., myelination, reductions in radial glial cells, and alterations in neuron morphology) and relate these to specific observations in diffusivity parameters. These findings further support the use of the pig as model for human brain development and highlight sensitive changes in neurodevelopment across a short time span. By characterizing these changes over time, we are able to better identify clinically relevant, critical windows of neurodevelopment when early-life nutrition may have the greatest influence.

## Conclusion

Using multiple imaging techniques, we were able to non-invasively characterize changes in brain development related to early-life dietary iron deficiency followed by dietary iron repletion. Measures of total brain volume at PND 61 suggest dietary iron repletion is able to compensate for delayed whole brain growth at PND 32; however, diffusion FA measures suggest dietary iron repletion was not able to correct myelination and tissue organization in specific brain regions by PND 61. Many previous studies have shown structural changes in brain development after a period of iron deficiency, and our study expands upon this work by identifying aspects of brain development, which do not appear to benefit from iron repletion later in life. Thus, these data highlight the importance of the critical window during which adequate dietary iron is necessary to ensure proper brain growth trajectories are established. Moreover, this research suggests possible heightened sensitivity of myelination to dietary iron status, and future work should seek to expand upon the mechanisms through which these changes develop and persist despite iron repletion.

## Ethics Statement

All animal and experimental procedures were in accordance with the National Research Council Guide for the Care and Use of Laboratory Animals and approved by the University of Illinois at Urbana-Champaign Institutional Animal Care and Use Committee.

## Author Contributions

AM, LK, and RD were involved in study design and implementation. All authors were involved in data acquisition, analysis, and interpretation. All authors read and approved the final version of this manuscript.

## Conflict of Interest Statement

The authors declare that the research was conducted in the absence of any commercial or financial relationships that could be construed as a potential conflict of interest.
