# Supplementary material for: Dietary Iron Repletion following Early-Life Dietary Iron Deficiency Does Not Correct Regional Volumetric or Diffusion Tensor Changes in the Developing Pig Brain
Source: Front Neurol. 2018 Jan 11;8:735. doi: 10.3389/fneur.2017.00735 (PMC5768607; doi:10.3389/fneur.2017.00735)

Supplemental Table 1. Absolute brain volumes (mm^3)[1]

Brain Region	CONT		ID		SEM	*P*-Value		
	PND 32	PND 61	PND 32	PND 61		Diet	Day	Diet × Day
Caudate	523^b	639^c	478^a	628^c	9.8	0.03	<0.001	0.01
Cerebellum	7911	10140	7290	9383	129.9	<0.001	<0.001	0.38
Cerebral Aqueduct	44	58	41	54	0.8	<0.001	<0.001	0.66
Corpus Callosum	474	584	431	563	9.7	0.01	<0.001	0.06
Cerebrospinal Fluid	14658	18702	12404	18619	858.7	0.13	<0.001	0.19
Fourth Ventricle	64	81	59	75	1.2	<0.01	<0.001	0.32
Grey Matter	43601^b	48502^c	39316^a	48541^c	890.9	0.03	<0.001	<0.01
Hypothalamus	305^b	375^c	275^a	363^c	5.0	<0.01	<0.001	0.01
Internal Capsule	1778^b	2197^c	1611^a	2116^c	31.5	<0.01	<0.001	0.03
Lateral Ventricle	609^b	756^c	556^a	731^c	12.0	0.01	<0.001	0.05
Left Cortex	19992	25002	18285	24234	414.8	0.02	<0.001	0.08
Left Hippocampus	579	740	524	687	12.6	<0.001	<0.001	0.90
Medulla	2317	2972	2163	2805	39.7	<0.001	<0.001	0.82
Midbrain	2163	2707	1962	2556	34.0	<0.001	<0.001	0.23
Olfactory Bulb	3049^b	3885^c	2726^a	3862^c	67.9	0.03	<0.001	0.01
Pons	1398	1762	1295	1685	19.6	<0.001	<0.001	0.29
Putamen-Globus Pallidus	447^b	546^c	404^a	535^c	8.1	0.01	<0.001	<0.01
Right Cortex	20684^b	25382^c	19013^a	24724^c	394.1	0.03	<0.001	0.02
Right Hippocampus	598	747	539	696	11.1	<0.001	<0.001	0.56
Thalamus	1715	2121	1544	2017	29.7	<0.001	<0.001	0.09
Third Ventricle	59	75	53	71	1.2	<0.01	<0.001	0.08
White Matter	22266	28772	22723	28358	754.5	0.98	<0.001	0.46
Whole Brain	72484^b	90493^c	66464^a	87647^c	1223.0	0.01	<0.001	0.02

[1]Data presented as mean and pooled standard error of the means (SEM) for each dietary treatment group. Main effects of dietary treatment (Diet; CONT vs ID) and MRI day (Day; PND 32 vs 61) and the interaction between Diet and Day are presented. [abc]Labeled means in a row without a common superscript letter differ, $P < 0.05$. Abbreviations: control (CONT), iron deficient (ID), postnatal day (PND).

Supplemental Table 2. Relative brain volumes (%TBV)[1]

Brain Region	CONT		ID		SEM	*P*-Value		
	PND 32	PND 61	PND 32	PND 61		Diet	Day	Diet × Day
Caudate	0.7218	0.7055	0.7190	0.7176	0.00598	0.49	0.05	0.10
Cerebellum	10.9262[ab]	11.2089[c]	10.9741[bc]	10.7240[a]	0.09691	0.07	0.78	<0.001
Cerebral Aqueduct	0.0611	0.0634	0.0615	0.0614	0.00088	0.37	0.16	0.11
Corpus Callosum	0.6535	0.6442	0.6484	0.6429	0.00448	0.48	0.07	0.62
Cerebrospinal Fluid	20.1640	20.8222	18.6594	21.1863	0.89650	0.42	0.09	0.31
Fourth Ventricle	0.0878	0.0893	0.0891	0.0859	0.00151	0.50	0.48	0.07
Grey Matter	60.1824	54.7462	59.1802	55.5527	0.93320	0.92	<0.001	0.21
Hypothalamus	0.4207	0.4138	0.4141	0.4144	0.00423	0.53	0.28	0.23
Internal Capsule	2.4537	2.4244	2.4246	2.4188	0.01203	0.18	0.09	0.24
Lateral Ventricle	0.8405	0.8344	0.8364	0.8352	0.00525	0.75	0.42	0.59
Left Cortex	27.5774	27.5932	27.5054	27.6602	0.17830	0.99	0.60	0.67
Left Hippocampus	0.7987	0.8146	0.7878	0.7834	0.00748	0.01	0.40	0.14
Medulla	3.2025	3.2731	3.2573	3.2098	0.05016	0.93	0.79	0.18
Midbrain	2.9874	2.9862	2.9540	2.9207	0.02401	0.07	0.34	0.37
Olfactory Bulb	4.2071[ab]	4.2856[bc]	4.1022[a]	4.4204[c]	0.06511	0.83	<0.001	0.03
Pons	1.9308	1.9457	1.9509	1.9272	0.02129	0.97	0.78	0.23
Putamen-Globus Pallidus	0.6167[b]	0.6032[a]	0.6086[ab]	0.6111[ab]	0.00418	0.98	0.07	0.01
Right Cortex	28.5211	28.0498	28.6020	28.2449	0.16000	0.37	0.01	0.70
Right Hippocampus	0.8247	0.8238	0.8110	0.7943	0.00612	<0.01	0.08	0.11
Thalamus	2.3676	2.3365	2.3248	2.3072	0.01621	0.04	0.09	0.63
Third Ventricle	0.08201	0.08253	0.0800	0.0861	0.00084	0.09	0.15	0.46
White Matter	30.7870	31.6419	34.2077	32.3016	0.95190	0.03	0.55	0.12

[1]Data presented as mean and pooled standard error of the means (SEM) for each dietary treatment group. Main effects of dietary treatment (Diet; CONT vs ID) and MRI day (Day; PND 32 vs 61) and the interaction between Diet and Day are presented. [abc]Labeled means in a row without a common superscript letter differ, $P < 0.05$. Abbreviations: control (CONT), iron deficient (ID), percent of total brain volume (%TBV), postnatal day (PND).

Supplemental Table 3. Radial diffusivity measures (×$10^{-3}mm^2/s$)[1]

Brain Region	CONT		ID			*P*-Value		
	**PND 32**	**PND 61**	**PND 32**	**PND 61**	**SEM**	**Diet**	**Day**	**Diet × Day**
Caudate	0.754	0.699	0.754	0.712	0.0120	0.60	<0.001	0.56
Cerebellum	1.020	1.000	1.040	0.999	0.0220	0.77	0.03	0.55
Corpus Callosum	1.100	1.020	1.080	1.010	0.0320	0.70	<0.001	0.84
Internal Capsule	0.628	0.603	0.637	0.612	0.0050	0.10	<0.001	0.98
Left Cortex	0.793	0.745	0.802	0.765	0.0060	0.03	<0.001	0.20
Left Hippocampus	0.891	0.872	0.905	0.888	0.0230	0.59	0.23	0.93
Right Cortex	0.775	0.754	0.800	0.779	0.0070	<0.001	<0.01	0.99
Right Hippocampus	0.926	0.878	0.892	0.866	0.0230	0.37	0.04	0.53
Thalamus	0.713	0.691	0.724	0.701	0.0070	0.08	<0.01	0.92
Whole Brain	0.827	0.772	0.848	0.804	0.0060	<0.01	<0.001	0.24

[1]Data presented as mean and pooled standard error of the means (SEM) for each dietary treatment group. Main effects of dietary treatment (Diet; CONT vs ID) and MRI day (Day; PND 32 vs 61) and the interaction between Diet and Day are presented. Abbreviations: control (CONT), iron deficient (ID), postnatal day (PND).

Supplemental Table 4. Mean diffusivity measures (×$\boldsymbol{10^{-3} mm^2/s}$)[1]

Brain Region	CONT		ID		SEM	*P* - Value		
	PND 32	PND 61	PND 32	PND 61		Diet	Day	Diet × Day
Caudate	0.930	0.865	0.921	0.878	0.0130	0.86	<0.001	0.35
Cerebellum	1.130	1.110	1.140	1.110	0.0230	0.93	0.11	0.63
Corpus Callosum	1.320	1.250	1.290	1.230	0.0370	0.57	0.01	0.76
Internal Capsule	0.844	0.833	0.847	0.837	0.0040	0.51	<0.01	0.84
Left Cortex	0.962	0.915	0.969	0.934	0.0060	0.07	<0.001	0.18
Left Hippocampus	1.070	1.040	1.090	1.060	0.0260	0.56	0.11	0.93
Right Cortex	0.950	0.929	0.976	0.954	0.0070	<0.001	<0.01	0.99
Right Hippocampus	1.110	1.050	1.060	1.050	0.0250	0.34	0.04	0.26
Thalamus	0.864	0.836	0.871	0.845	0.0070	0.22	<0.01	0.95
Whole Brain	0.999	0.942	1.021	0.974	0.0070	<0.01	<0.001	0.28

[1]Data presented as mean and pooled standard error of the means (SEM) for each dietary treatment group. Main effects of dietary treatment (Diet; CONT vs ID) and MRI day (Day; PND 32 vs 61) and the interaction between Diet and Day are presented. Abbreviations: control (CONT), iron deficient (ID), postnatal day (PND).

Supplemental Table 5. Axial diffusivity measures ($\times$***10^{-3}mm^2/s***)[1]

Brain Region	**CONT**		**ID**			***P*-Value**		
	**PND 32**	**PND 61**	**PND 32**	**PND 61**	**SEM**	**Diet**	**Day**	**Diet × Day**
Caudate	1.283	1.195	1.255	1.212	0.0170	0.70	<0.01	0.17
Cerebellum	1.344	1.342	1.341	1.328	0.0240	0.76	0.66	0.77
Corpus Callosum	1.753	1.703	1.695	1.673	0.0470	0.42	0.25	0.66
Internal Capsule	1.276	1.289	1.265	1.288	0.0060	0.34	<0.01	0.41
Left Cortex	1.301	1.255	1.303	1.273	0.0070	0.20	<0.001	0.15
Left Hippocampus	1.431	1.390	1.462	1.407	0.0330	0.52	0.05	0.77
Right Cortex	1.306	1.281	1.326	1.303	0.0090	0.01	<0.01	0.91
Right Hippocampus	1.488	1.399	1.413	1.407	0.0300	0.32	0.04	0.07
Thalamus	1.167	1.125	1.167	1.132	0.0120	0.71	<0.01	0.82
Whole Brain	1.342	1.282	1.366	1.315	0.0080	<0.01	<0.001	0.36

[1]Data presented as mean and pooled standard error of the means (SEM) for each dietary treatment group. Main effects of dietary treatment (Diet; CONT vs ID) and MRI day (Day; PND 32 vs 61) and the interaction between Diet and Day are presented. Abbreviations: control (CONT), iron deficient (ID), postnatal day (PND).

Supplemental Figure 1. Fractional anisotropy values generated from T1-weighted white matter image

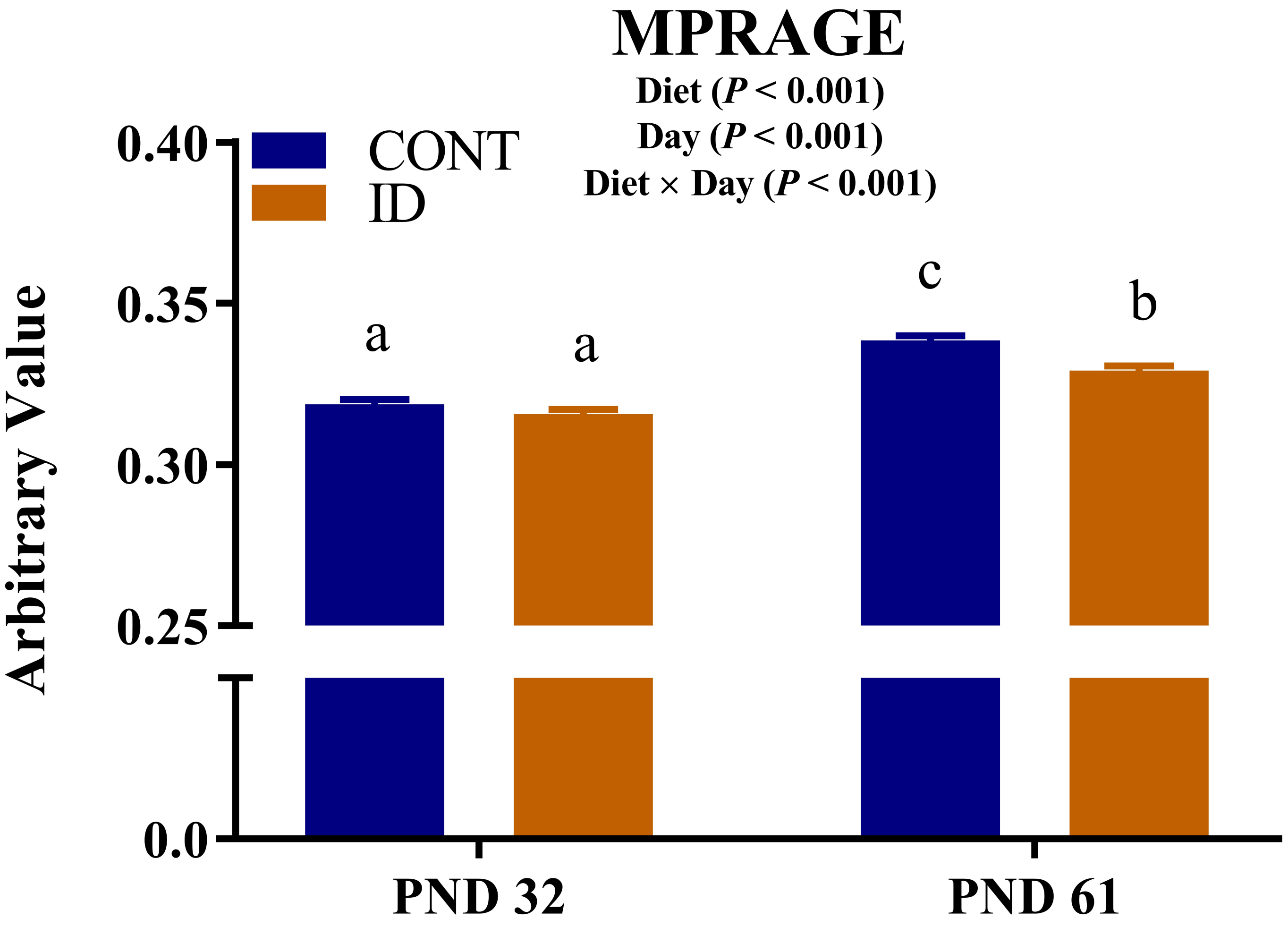

Supplement: Supplementary file 1 [file Data_Sheet_1.PDF]
